# Selection of parental lines for plant breeding *via* genomic prediction

**DOI:** 10.3389/fpls.2022.934767

**Published:** 2022-07-27

**Authors:** Ping-Yuan Chung, Chen-Tuo Liao

**Affiliations:** ^1^Department of Agronomy, National Taiwan University, Taipei, Taiwan; ^2^Institute of Statistical Science, Academia Sinica, Taipei, Taiwan

**Keywords:** genetic gain, genome-wide markers, mixed models, multiple-trait selection, selection index

## Abstract

A set of superior parental lines is imperative for the development of high-performing inbred lines in any biparental crossing program for crops. The main objectives of this study are to (a) develop a genomic prediction approach to identify superior parental lines for multi-trait selection, and (b) generate a software package for users to execute the proposed approach before conducting field experiments. According to different breeding goals of the target traits, a novel selection index integrating information from genomic-estimated breeding values (GEBVs) of candidate accessions was proposed to evaluate the composite performance of simulated progeny populations. Two rice (*Oryza sativa* L.) genome datasets were analyzed to illustrate the potential applications of the proposed approach. One dataset applied to the parental selection for producing inbred lines with satisfactory performance in primary and secondary traits simultaneously. The other one applied to demonstrate the application of producing inbred lines with high adaptability to different environments. Overall, the results showed that incorporating GEBV and genomic diversity into a selection strategy based on the proposed selection index could assist in selecting superior parents to meet the desired breeding goals and increasing long-term genetic gain. An R package, called IPLGP, was generated to facilitate the widespread application of the approach.

## Introduction

Parental line selection in plant breeding usually has two differing goals: (i) identify suitable parents for commercial hybrid varieties and (ii) identify suitable parents to develop inbred lines for subsequent breeding cycles (Gaynor et al., 2017). For goal (i), the selection is based on an evaluation of hybrid performance (Wu et al., 2019); however, for goal (ii), the selection is based on the performance of progeny populations (Chung and Liao, 2020). In this study, we focus on the latter, i.e., parental line selection for the development of high-performing inbred lines using a biparental crossing scheme. Genomic selection based on the statistical method of genomic prediction (GP) has emerged as a promising approach to improving quantitative traits. The main concept of GP is to capture all the effects of quantitative trait loci by using high-density DNA markers over an entire genome (Meuwissen et al., 2001). Marker effects are estimated using a GP model built from phenotypic and genotypic data of a training population. After model training, genomic estimated breeding values (GEBVs) for candidate accessions are estimated from their genotypic data alone. Genomic selection is then performed based on these resulting GEBVs (Heffner et al., 2010).

Because genomic selection was specifically designed to predict complex traits such as the grain yield (YLD) of a crop, most published genomic selection studies have focused on single-trait approaches without exploiting information from multiple correlated agronomic traits (Schulthess et al., 2016). Yet, attaining most breeding goals usually requires the improvement of multiple traits. Besides a high YLD, an ideal cultivar is also expected to perform well in some secondary traits (Guo et al., 2020; Sandhu et al., 2021). For example, in rice, a low plant height (PH) can reduce the incidence of lodging and an early flowering time (FT) can reduce the cultivation period; therefore, simultaneously inheriting these traits of high YLD, low PH, and early FT is often sought by rice breeders. In practice, it would be desirable to develop appropriate GP approaches for multi-trait genomic selection (MTGS). In studies by Jia and Jannink (2012); Hayashi and Iwata (2013), and Guo et al. (2014), they reported that prediction accuracy for a target trait with low heritability could be substantially improved when a correlated indicator trait with higher heritability was also included in the GP model. There are three types of multi-trait GP models commonly used for MTGS, including (i) linear mixed models (VanRaden, 2008; Endelman, 2011), (ii) Bayesian models (Perez and de los Campos, 2014; Montesinos-Lopez et al., 2016), and (iii) machine- and deep-learning models (Smith et al., 2013; Lecun et al., 2015). Recently, Sandhu et al. (2021) compared the performance of the above models based on the prediction for grain yield and grain protein content in wheat (*Triticum aestivum* L.). The results of this article showed that multi-trait machine- and deep-learning models were able to increase prediction accuracy and should be employed in large-scale breeding programs. To harness the benefits of MTGS for plant breeding, Schulthess et al. (2016); Fernandes et al. (2018); Ward et al. (2019), and Guo et al. (2020) used multi-trait prediction models to augment quantitative traits in various crops.

A selection index is often used in multi-trait breeding programs because it combines information from multiple traits, and incorporates the capacity of favorable levels of some traits to compensate for unfavorable levels in other traits (Dolan et al., 1996). Several different selection indices can be used in MTGS, including those based on economic values, phenotypic correlations, genotypic correlations, and enhancing some traits while limiting other traits (Baker, 1986; Ceron-Rojas and Crossa, 2021). Schulthess et al. (2016) compared the prediction accuracies of different selection indices using various prediction methods and recommended implementing a single-trait GP model by treating a selection index itself as a new single trait. Covarrubias-Pazaran et al. (2018) demonstrated that the use of multi-trait genomic best linear unbiased prediction (multi-trait GBLUP) models could improve selection accuracy and subsequently lead to more reliable selection indices. Notably, those studies cited above focused on prediction accuracy for target traits or selection indices. Alternatively, Lehermeier et al. (2017) emphasized that genetic gain can be increased considerably when the crosses are selected based on their genomic usefulness function compared to selection based on mean GEBVs. In this respect, Yao et al. (2018) combined GP with Monte Carlo simulations to select superior parents for wheat breeding. The authors applied a selection index to incorporate YLD and two crop quality-related traits, and calculated a usefulness function based on the selection index values of simulated progeny populations. Their findings also showed that utilizing the usefulness function for parental selection is capable of providing higher genetic gain than the use of a mid-parent GEBV.

Both Lehermeier et al. (2017) and Yao et al. (2018) cautioned that parental selection strategies should not focus solely upon truncation selection that selects the top fraction of candidate accessions with the top GEBVs. To preserve genetic variation to maximize selection responses in progeny populations, plant breeders should avoid selecting closely related parental lines in the base population. Accordingly, Chung and Liao (2020) proposed strategies whereby both GEBV and genomic diversity (GD) were taken into account for single-trait selection. However, such single-trait selection strategies can result in different choices of parental lines for different target traits, and this may cause confusion in practical applications. The improvement of genetic stocks usually warrants considering multiple traits at once, because economic value and net genetic merits depend on almost all the traits responsible for the desired crop phenotype (Falconer and Mackay, 1996).

In this study, our aim was to develop and validate a useful genomic prediction approach to select parental lines for producing progeny populations with superior performance in multiple target traits. To do this, a multi-trait GBLUP model was used to simultaneously predict normalized GEBVs of the multiple target traits. A new selection index integrating information from the normalized GEBVs was then proposed to evaluate the composite performance of simulated progeny populations. Three different strategies considering GEBV and/or GD were compared through a stochastic simulation approach for producing progeny populations. Finally, an R package called IPLGP (Chung and Liao, 2022) was generated in conducting this study.

## Materials and methods

**Tropical rice genome dataset:** The rice (*Oryza sativa* L.) genome dataset presented in Spindel et al. (2015) was analyzed first. This dataset contains 73,147 single-nucleotide polymorphism (SNP) markers and 363 elite breeding lines belonging to *indica* or *indica-admixed* groups. The phenotypic data include 4 years (from 2009 to 2012), two seasons per year (dry and wet), and YLD, PH, and FT for each season. Unfortunately, PH data for the 2009 wet season were not available. Phenotypic values of 35 of the 363 breeding lines were also missing; hence, adjusted means derived from 328 breeding lines were used in our study. The adjusted means were obtained using the residuals derived separately for each trait by the following model:


(1) $$\begin{array}{l} y_{ijk}=\mu +A_{i}+S_{j}+{\left(AS\right)}_{ij}+B_{k}+e_{ijk} \end{array}$$

where *y*_*ijk*_ is the phenotypic value of the trait at year *i*, season *j* and block *k*; *A*_*i*_ is the fixed effect of year *i*; *S*_*j*_ is the fixed effect of season *j*; (*AS*)_*ij*_ is the interaction effect between year *i* and season *j*; *B*_*k*_ is the fixed effect of block *k*; and *e*_*ijk*_ is the residual. One SNP marker was randomly chosen per 0.1-cM interval over each chromosome because Spindel et al. (2015) had shown that the subset of the full markers was efficient enough for genomic selection for this collection of rice germplasm. This resulted in 10,772 out of the 73,147 SNP markers being used for this example. The SNP genotype at each locus was coded as −1, 0, or 1, where 1 indicates homozygosity for the major allele, −1 indicates homozygosity for the minor allele, and 0 indicates heterozygosity. After the SNP coding, any missing loci were imputed as 1.

**44k rice genome dataset:** The rice genome dataset is presented in Zhao et al. (2011). It was originally collected for a genome-wide association study and was reanalyzed here. It contains 44,100 SNP markers and 36 traits of 413 accessions, and this dataset features a strong subpopulation structure. All SNP markers with a missing rate > 0.05 and a minor allele frequency <0.05 were first removed from the dataset. This left 34,233 SNP markers. To avoid redundant SNP markers in calculating the genomic relationship between individuals, about one-third of these SNP markers (11,043 out of the 34,233) evenly distributed over each chromosome were selected. Their SNP coding was performed as described above for the tropical rice dataset. Only those 300 of the 413 accessions with no missing FT data from all the three locations—Arkansas (FT-Ark), Faridpur (FT-Far), and Aberdeen (FT-Abe)—were used here for building the required multi-trait GBLUP model. To simulate the genotypic data of progeny populations for both the rice datasets, the Gramene Annotated Nipponbare Sequence provided by Youens-Clark et al. (2011) was used to estimate recombination rates between two adjacent SNP markers.

### The multi-trait GBLUP model for fitting normalized phenotypic values

The target traits of interest were classified into three types according to their breeding goals. The *larger-the-better*: the larger phenotypic value is desirable; the *smaller-the-better*: the smaller phenotypic value is desirable; and the *nominal-the-best*: the nominal value is the best because it is the one that satisfies the target set by the plant breeder. Therefore, a given phenotypic value that falls around the nominal value is desirable for this last type. For example, FT may be set to a specific time for balancing the duration of cultivation and the vegetative growth period. Accordingly, the vectors of phenotypic values for traits were first normalized as follows. Let ***w***_*i*_ = (***y***_*i*_ − δ**1**_*n*_)/*s*_*i*_, where δ is set to the sample mean of the phenotypic values for both the larger-the-better and the smaller-the-better types, and to the desired target value for the nominal-the-best type; *s*_*i*_ is the sample standard deviation of those phenotypic values; **1**_*n*_ is the vector of order *n* with all elements equal to 1; and $y_{i}={\left[y_{i1},\;\ldots ,\;y_{in}\right]}^{T}$ is the vector of phenotypic values for *i* = 1, 2, …, *t*. Here *n* is the number of individuals in the training population, and *t* is the number of target traits.

Let


$$\begin{array}{l} \text{w}=\left[\begin{array}{c} {\text{w}}_{1}\\ \vdots \\ {\text{w}}_{t} \end{array}\right];\mu =\left[\begin{array}{c} \mu_{1}\\ \vdots \\ \mu_{t} \end{array}\right];\;\text{g}=\left[\begin{array}{c} {\text{g}}_{1}\\ \vdots \\ {\text{g}}_{t} \end{array}\right];\text{and }\text{e}=\left[\begin{array}{c} {\text{e}}_{1}\\ \vdots \\ {\text{e}}_{t} \end{array}\right] \end{array}$$

where μ_*i*_, ***g***_*i*_, and ***e***_*i*_, respectively, denote the general mean, the vector of genomic values, and the vector of random errors for trait *i*. The additive effects multi-trait GBLUP model is given as:


(2) $$\begin{array}{l} \text{w}=\mu ⊗1_{n}+\text{g}+\text{e} \end{array}$$

where ⊗ denotes the Kronecker product (Searle, 1982). It is assumed that ***g*** and ***e*** are mutually independent and separately follow a multivariate normal distribution, as denoted by


$$\begin{array}{l} \text{g}\;\sim \;MVN\left(0,{\text{Σ}}_{\text{A}}⊗\text{K}\right)\; \end{array}$$

and


$$\begin{array}{l} \;\text{e}\sim \;MVN\left(0,{\text{Σ}}_{\text{e}}⊗{\text{I}}_{n}\right)\; \end{array}$$

where **0** is a zero vector, **Σ**_***A***_ is the genetic variance-covariance matrix for additive effects among the *t* target traits, ***K*** is a genomic relationship matrix for additive effects among the *n* individuals, **Σ**_***e***_ is the residual variance-covariance matrix among the *t* target traits, and ***I***_*n*_ is the identity matrix of order *n*. Here, **Σ**_***A***_ and **Σ**_***e***_ can be represented as


$$\begin{array}{l} {\text{Σ}}_{A}=\left[\begin{array}{ccc} \sigma_{A_{1}}^{2} & \cdots & \sigma_{A_{1t}}\\ \vdots & \ddots & \vdots \\ \sigma_{A_{t1}} & \cdots & \sigma_{A_{t}}^{2} \end{array}\right]\text{ and }{\text{Σ}}_{e}=\left[\begin{array}{ccc} \sigma_{e_{1}}^{2} & \cdots & \sigma_{e_{1t}}\\ \vdots & \ddots & \vdots \\ \sigma_{e_{1t}} & \cdots & \sigma_{e_{t}}^{2}\\ \; \end{array}\right] \end{array}$$

where $\sigma_{A_{i}}^{2}$ and $\sigma_{e_{i}}^{2}$ are the respective variances for the additive effects and the random errors for trait *i*, and σ_*A*_*ij*__ and σ_*e*_*ij*__ are the corresponding covariances between traits *i* and *j*. The genomic relationship matrix was calculated as ***K* = *MM***^*T*^/*p*, where ***M*** is the marker coding matrix regarding the additive effects, and *p* is the number of markers.

Let $\hat{\mu }$ be the best linear unbiased estimate (BLUE) for **μ**, and $\hat{g}$ be the best linear unbiased predictor (BLUP) for ***g***, then $\hat{\mu }$ and $\hat{g}$ can be obtained from the following linear mixed model equations (Henderson, 1975):


(3) $$\begin{array}{l} \left[\begin{array}{cc} nI_{t} & I_{t}⊗1_{n}^{T}\\ I_{t}⊗1_{n} & I_{nt}+{(\text{Σ}}_{e}{{\text{Σ}}_{A}}^{-1})⊗K^{-1} \end{array}\right]\left[\begin{array}{c} \hat{\mu }\\ \hat{g} \end{array}\right]=\left[\begin{array}{c} \left(I_{t}⊗1_{n}^{T}\right)w\\ w \end{array}\right]\text{. } \end{array}$$

The restricted maximum likelihood estimates (REMLs) for **Σ_*A*_** and **Σ_*e*_** were plugged into Eq. (3) to generate $\hat{\mu }$ and $\hat{g}.$ The R package *sommer* (Covarrubias-Pazaran, 2016) was used to calculate these estimates from training data.

### Predicting GEBVs for simulated progeny populations

The performance of a set of parental lines was evaluated based on the GEBVs of their progeny populations. Genotypic data of the progeny populations were generated using the simulation approach of Chung and Liao (2020). This was mainly based on the mapping function of recombination rate on linkage distance between two adjacent markers as presented in Haldane (1919). The required GEBVs were then predicted using the multi-trait GBLUP model of (2). Let ***h***_*i*_ denote the vector of genomic values for trait *i* in a simulated progeny population, and ***K***_*pt*_ denote the genomic relationship matrix between the simulated progeny population and the training population. From Henderson (1977), the BLUP for ***h***_*i*_ is given by


(4) $$\begin{array}{l} {\hat{h}}_{i}\;=\;K_{pt}K^{-1}\;{\hat{g}}_{i}\; \end{array}$$

where ${\hat{g}}_{i}$ is the BLUP for the vector of genomic values of trait *i* obtained from Eq. (3). The GEBVs for the simulated progeny population are then predicted by ${\hat{\mu }}_{i}1_{n}+{\hat{h}}_{i}$, where ${\hat{\mu }}_{i}$ is the BLUE of μ_*i*_ obtained from Eq. (3), for *i* = 1, 2, …, *t*.

### The selection index

For a particular individual, the selection index below was used to integrate its normalized GEBVs for the multiple target traits:


(5) $$\begin{array}{l} \text{SI}=\sum_{\text{i}=1}^{\text{t}}{\text{w}}_{\text{i}}Z_{i}\; \end{array}$$

where *w*_*i*_ is a pre-specified weight for trait *i* subject to the constraint that $\overset{t}{\underset{i=1}{\sum }}w_{i}=1$; and *Z*_*i*_ is designated as *GEBV*_*i*_ for the larger-the-better case, as −*GEBV*_*i*_ for the smaller-the-better case, and as −|*GEBV*_*i*_| (the absolute value of *GEBV*_*i*_) for the nominal-the-best case. The selection index conveys an overall performance score for the individual. Note that the normalized *GEBV*_*i*_ are scalars with no measuring units. The larger the selection index, the better the composite performance.

### Procedure for selecting superior parental lines

For the tropical rice dataset, the aim was to select a set of parental lines whose progeny populations had high YLD, low PH, and low FT. For the 44k rice dataset, the breeding goal was assumed to identify a set of superior accessions that would produce inbred lines with an FT as close as possible to the nominal value set as 80 days at all three locations (FT-Ark, FT-Far, and FT-Abe). The resulting inbred lines would be anticipated to have high adaptability to the three different locations. The selection procedure can be described as follows.

#### Step 1

All available phenotypic values in each dataset were normalized as described above. The ensuing normalized data were used to build the multi-trait GBLUP model given by Eq. (2). The trained multi-trait GBLUP model predicted the normalized GEBVs of the target traits for each dataset; then the corresponding selection index values were obtained for all the accessions in the candidate population.

The selection index integrating the normalized GEBVs of the three target traits in the tropical rice dataset was defined this way:


(6) $$\begin{array}{l} \text{SI}\left(\text{tropical}\right)={\text{w}}_{1}\text{GEB}{\text{V}}_{\text{YLD}}-{\text{w}}_{2}\text{GEB}{\text{V}}_{\text{PH}}-{\text{w}}_{3}\text{GEB}{\text{V}}_{\text{FT}} \end{array}$$

where *w*_1_, *w*_2_, and *w*_3_ are pre-specified index weights. Note the minus signs applied in the equation for PH and FT because smaller values are preferable for these two traits. The index weights *w*_1_, *w*_2_, and *w*_3_ were respectively specified as 0.6, 0.2, and 0.2. For the sake of contrast, another setting of 1, 0, and 0 was used that corresponded to the single-trait selection for YLD. To compare the improvement of the strategy changed from single-trait selection to multi-trait selection, an index was defined as follows:


(7) $$\begin{array}{l} \text{IR}=\left[\text{GEBV of }\left(0\text{.}6,\;0\text{.}2,\;0\text{.}2\right)-\text{GEBV of }\left(1,\;0,\;0\right)\;\right]\\ \;÷\text{GEBV of }\left(1,\;0,\;0\right)\times 100\%\text{. } \end{array}$$

The selection index for the 44k rice dataset was defined as:


(8) $$\begin{array}{l} \text{SI}\left(44\text{k}\right)=-w_{1}|GEBV_{FT-Ark}|-w_{2}|GEBV_{FT-Far}|\\ \;-w_{3}{|GEBV}_{FT-Abe}| \end{array}$$

where the index weights *w*_1_, *w*_2_, and *w*_3_ were equally set to be 1/3.

#### Step 2

Based on the normalized GEBVs of the candidate accessions obtained from Step 1, three strategies were implemented to select a subset of 10 parental lines from the candidate population. (i) The GEBV only (GEBV-O) strategy, which selected the top 10 accessions with the highest selection index values. (ii) The GD only (GD-O) strategy, which searched for an optimal subset of 10 accessions from *S*_*c*_ that is the set composed of those accessions whose selection index values were above average. This resulting optimal subset achieved the maximal *D-*score, where the *D-*score is the determinant of the genomic relationship matrix corresponding to the selected accessions, and it was used to measure the genomic diversity of the selected accessions (Chung and Liao, 2020). (iii) The GEBV-GD strategy, which considered both GEBV and GD. This strategy retained the top two accessions with the highest selection index values, and then searched for another eight accessions among the remainder of *S*_*c*_. The resulting 10 accessions achieved the maximal *D*-score.

#### Step 3

For each subset of 10 parental lines generated from Step 2, any two parental lines were crossed to produce 45 F_1_ hybrids, and then each F_1_ hybrid produced 60 individuals by self-pollinating and applying the simulation approach of Chung and Liao (2020); hence, a total of 45 × 60 = 2, 700 F_2_ individuals. Again, the GEBVs of F_2_ individuals were calculated *via* the trained multi-trait GBLUP models in Step 1. The individuals with the top 45 selection index values in the F_2_ generation were selected, and those produced 2,700 F_3_ individuals (each F_2_ individual produced 60 F_3_ individuals).

#### Step 4

The procedure of generating genotypic data, predicting the GEBVs, and selecting the top 45 individuals was performed repeatedly, to produce 2,700 F_10_ individuals presumed to constitute a fixed population of inbred lines.

#### Step 5

For the final 2,700 F_10_ individuals generated from each subset of 10 parental lines, the best F_10_ inbred line with the highest index value was identified. The above analysis procedure was repeated 30 times to obtain the best F_10_ inbred lines from each repetition per strategy. To evaluate improvements in both the larger-the-better and the smaller-the-better target traits attained by each strategy, the genetic gain was calculated this way:


(9) $$\begin{array}{l} \text{genetic gain}={\bar{GEBV}}_{F_{10}}-{\bar{GEBV}}_{P}\; \end{array}$$

where ${\bar{GEBV}}_{F_{10}}$ is the GEBV average among the 2,700 F_10_ individuals, and ${\bar{GEBV}}_{P}$ is the GEBV average among the 10 selected parental lines. For the nominal-the-best target trait, genetic gain was defined as follows:


(10) $$\begin{array}{r} \text{genetic gain }\left(\text{nominal}\right)=mean\;\left(\left|GEBV_{F_{10}}-\delta \right|\right)\\ -mean\left(\left|GEBV_{p}-\delta \right|\right)\; \end{array}$$

where *mean*(|*GEBV*_*F*_10__ − δ|) is the averaged deviation of the F_10_ inbred lines from the nominal value δ, and *mean*(|*GEBV*_*p*_ − δ|) is the averaged deviation of the parental lines from δ.

The GEBVs of the target traits on their original measuring units were obtained *via* back-transformation from their normalized GEBVs. Furthermore, pairwise comparisons among the GEBV averages for each trait were carried out using the least significant difference (LSD) test.

## Results

Hereafter, GEBVs are reported for the target traits based on their original scales.

### Tropical rice genome dataset

**Figure 1:**
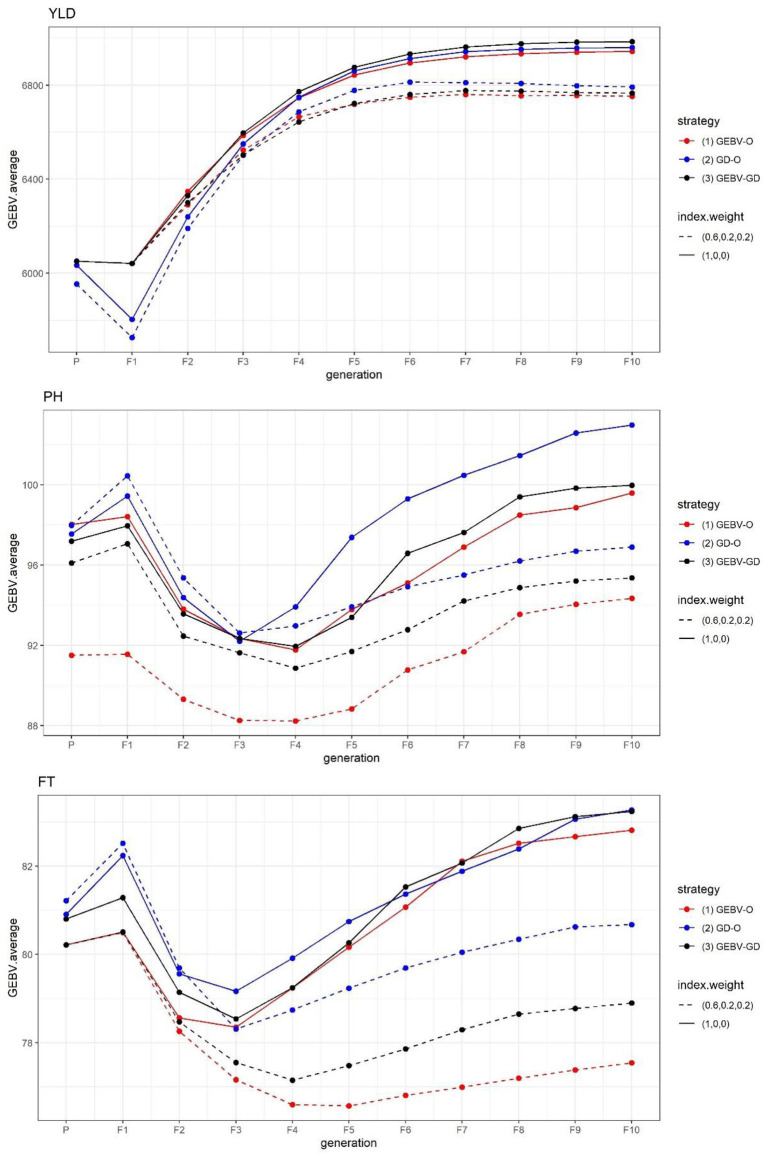
GEBV averages of the best individuals at each generation for the tropical rice dataset. GEBV-O, Subset of the 10 accessions with the highest selection index values; GD-O, Subset of the 10 accessions with the maximal *D*-scores chosen from the candidate set *S*_*c*_; GEBV-GD, Subset of the top two accessions with the highest selection index values, and another eight accessions chosen from the reminder of *S*_*c*_. YLD, grain yield; PH, plant height; FT, flowering time.

The GEBV averages of the best individuals from the 30 repetitions per generation are displayed in Figure 1. Evidently, the sought-after GEBV average decreased from parental generation to F_1_ generation. In contrast to YLD, which improved from F_1_ down through the F_10_ generation under every strategy tested, the desirability in the GEBV average for both PH and FT improved going from the F_1_ to F_3_ or F_4_ generation, but gradually declined in later generations. A strategy with the index weight of 0.2 provided the best F_10_ inbred lines, these having a better PH and FT than those generated from the same strategy whose index weight was 0.

The end-point of the GEBV averages for the best F_10_ individuals from the 30 repetitions and the improvement of Eq. (7) are presented in Table 1. For any of the three strategies GEBV-O, GD-O, and GEBV-GD, using selection index weights of 1, 0, 0 (i.e., the single-trait selection for YLD) always outperformed 0.6, 0.2, and 0.2 for YLD in terms of statistical significance. Conversely, any strategy with selection index weights of 0.6, 0.2, and 0.2 led to greater improvement in the two secondary target traits PH and FT than when using 1, 0, and 0 instead. The GEBV-GD had the largest GEBV average for YLD among the three strategies when using index weights of 1, 0, and 0, and the GD-O had the largest one when 0.6, 0.2, and 0.2 index weights were used. However, for YLD, there was no significant difference among the three strategies when applying either set of index weights. The GD-O performed worst with respect to GEBV averages for both PH and FT among the three strategies, with index weights of 0.6, 0.2, and 0.2. For the GEBV-O strategy, the secondary traits of PH and FT were respectively improved by 5.27 and 6.36%, but the primary trait of YLD fell by 2.83%, when the index weights 1, 0, and 0 were changed to 0.6, 0.2, and 0.2. The improvements obtained under GD-O amounted to 5.90% (gain in PH), 3.12% (gain in FT), and 2.85% (loss in YLD). Moreover, corresponding percentages for the GEBV-GD were 4.62% (gain in PH), 5.21% (gain in FT), and 3.13% (loss in YLD). Consequently, for this dataset, either GEBV-GD or GEBV-O with index weights of 0.6, 0.2, or 0.2 may be used to select a suitable set of parental lines for producing high-performing inbred lines simultaneously featuring high YLD, low PH, and low FT traits.

**Table 1:** GEBV averages of the best F_**10**_ inbred lines for the tropical rice dataset.

			**Traits** ^2^	
**Strategy** ^1^	**Index weights**	**YLD**	**PH**	**FT**
GEBV-O	(1, 0, 0)	6,943.55^a^	99.59^c^	82.81^d^
	(0.6, 0.2, 0.2)	6,752.50^b^	94.34^a^	77.54^a^
	IR (%)	−2.85	−5.27	−6.36
GD-O	(1, 0, 0)	6,960.07^a^	102.98^d^	83.27^d^
	(0.6, 0.2, 0.2)	6,791.71^b^	96.90^b^	80.67^c^
	IR (%)	−2.42	−5.90	−3.12
GEBV-GD	(1, 0, 0)	6,984.92^a^	99.98^c^	83.24^d^
	(0.6, 0.2, 0.2)	6,766.03^b^	95.36^ab^	78.90^b^
	IR (%)	−3.13	−4.62	−5.21

1 *GEBV-O, Subset of the 10 accessions with the highest selection index values; GD-O, Subset of the 10 accessions with the maximal D-scores chosen from the candidate set S_c_; GEBV-GD, Subset of the top two accessions with the highest selection index values, and another eight accessions chosen from the reminder of S_c_.*

2 *YLD, grain yield; PH, plant height; FT, flowering time.*

*Different lowercase letters indicate significant differences between the strategies for a given trait (p < 0.01; LSD test).*

*IR = [GEBV of (0.6, 0.2, 0.2)−GEBV of (1, 0, 0)] ÷ GEBV of (1, 0, 0) × 100%*.

The average genetic gain for a given target trait, as calculated by Eq. (9), from the 30 repetitions appears in Table 2. Based on these results, the ${\bar{GEBV}}_{P}$ can be ranked as GEBV-O > GEBV-GD > GD-O in descending desirability when using selection index weights of 0.6, 0.2, 0.2 or 1, 0, 0. The ranking of genetic gains is reversed to GD-O > GEBV-GD > GEBV-O with any of the two index weights used for all the three target traits, except that GEBV-GD > GD-O > GEBV-O ensues with index weights of 1, 0, and 0 for PH. Nevertheless, the genetic gain in PH from GEBV-GD (−8.25 cm) did not differ significantly from GD-O (-7.73 cm). Any strategy applied had a greater genetic gain in YLD with index weights of 1, 0, and 0 than 0.6, 0.2, and 0.2. Conversely, any strategy had a greater genetic gain on both PH and FT with index weights of 0.6, 0.2, and 0.2 than 1, 0, and 0, except in the case of GEBV-O upon PH (the former had −3.27 cm and the latter had −5.59 cm).

**Table 2:** GEBV average for parental lines, GEBV average for F_**10**_ inbred lines, and genetic gain for the tropical rice dataset.

**Strategy** ^1^	**Index weights**	${\bar{GEBV}}_{p}$	${\bar{GEBV}}_{F_{10}}$	**Genetic gain** ^2^
**YLD (grain yield)**				
GEBV-O	(1, 0, 0)	5,772.16	6,936.65	1,164.79^d^
	(0.6, 0.2, 0.2)	5,667.37	6,742.90	1,075.53^e^
GD-O	(1, 0, 0)	5,250.25	6,954.57	1,704.33^a^
	(0.6, 0.2, 0.2)	5,223.77	6,782.19	1,558.41^b^
GEBV-GD	(1, 0, 0)	5,575.64	6,979.08	1,403.43^c^
	(0.6, 0.2, 0.2)	5,561.36	6,759.18	1,197.81^d^
**PH (plant height)**				
GEBV-O	(1, 0, 0)	105.47	99.89	−5.59^d^
	(0.6, 0.2, 0.2)	97.84	94.57	−3.27^e^
GD-O	(1, 0, 0)	111.00	103.27	−7.73^cd^
	(0.6, 0.2, 0.2)	111.33	97.17	−14.17^a^
GEBV-GD	(1, 0, 0)	108.52	100.27	−8.25^c^
	(0.6, 0.2, 0.2)	106.14	95.50	−10.64^b^
**FT (flowering time)**				
GEBV-O	(1, 0, 0)	84.54	82.96	−1.58^e^
	(0.6, 0.2, 0.2)	82.29	77.63	−4.66^c^
GD-O	(1, 0, 0)	87.79	83.40	−4.39^c^
	(0.6, 0.2, 0.2)	87.97	80.81	−7.16^a^
GEBV-GD	(1, 0, 0)	86.15	83.43	−2.72^d^
	(0.6, 0.2, 0.2)	84.55	78.98	−5.57^b^

1 *GEBV-O, Subset of the 10 accessions with the highest selection index values; GD-O, Subset of the 10 accessions with the maximal D-scores chosen from the candidate set S_c_; GEBV-GD, Subset of the top two accessions with the highest selection index values, and another eight accessions chosen from the reminder of S_c_.*

2 *Different lowercase letters indicate significant differences among the strategies for a given trait (p < 0.01; LSD test)*.

### 44k rice genome dataset

**Figure 2:**
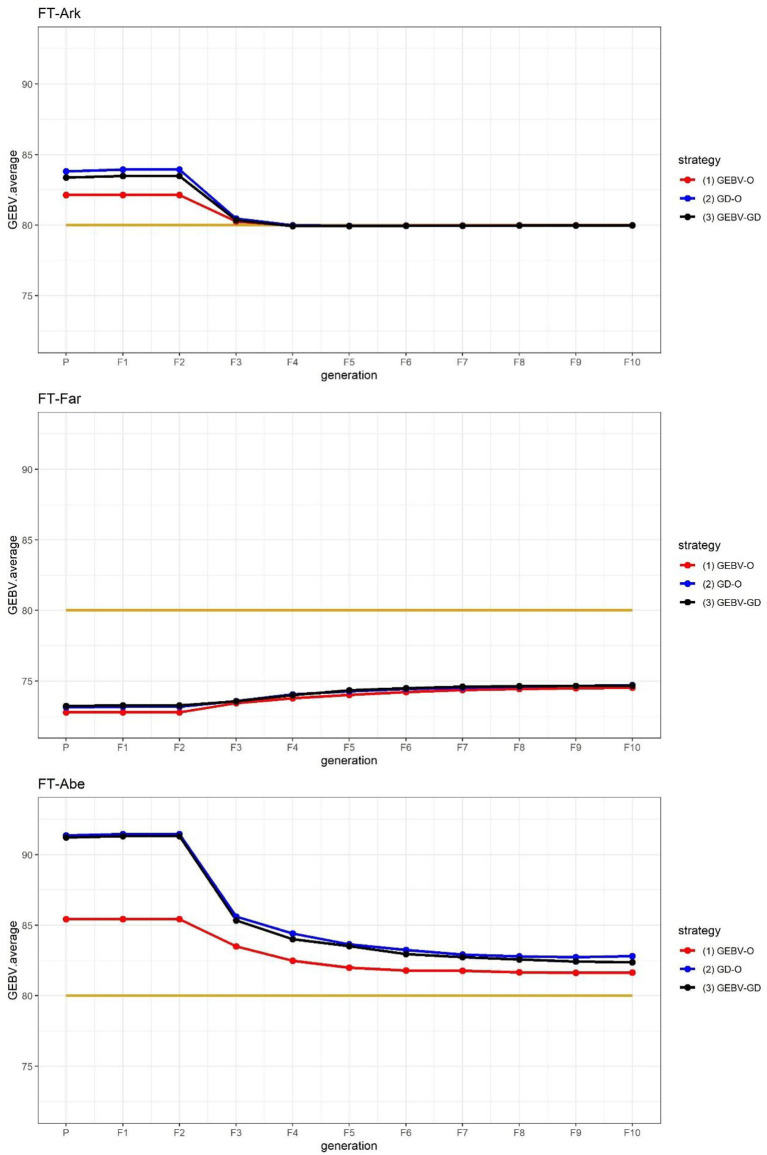
GEBV averages of the best individuals at each generation for the 44k rice dataset based on the index weights of 1/3, 1/3, and 1/3. GEBV-O, Subset of the 10 accessions with the highest selection index values; GD-O, Subset of the 10 accessions with the maximal *D*-scores chosen from the candidate set *S*_*c*_; GEBV-GD, Subset of the top two accessions with the highest selection index values, and another eight accessions chosen from the reminder of *S*_*c*_. FT-Ark, flowering time in Arkansas; FT-Far, flowering time in Faridpur; FT-Abe, flowering time in Aberdeen.

The GEBV averages of the best individuals from the 30 repetitions per generation are shown in Figure 2, for which the two GEBV averages of the parental and F_10_ generations are in Table 3. From Figure 2, it is interesting to see that all the curves approached the nominal value of 80 days. From Table 3, the three end-point values of ${\bar{GEBV}}_{F_{10}}$ for ${\bar{GEBV}}_{F_{10}}$ for FT-Far are about 74 days, this slightly less ${\bar{GEBV}}_{F_{10}}$ values of FT-Far differed significantly from those of FT-Ark and FT-Abe, and the FT-Far seems less improved than either FT-Ark or FT-Abe. At these three locations, based on the LSD testing for ${\bar{GEBV}}_{F_{10}}$, the GEBV-O, or GD-O implemented with equal index weights can be used to select a set of parental lines for producing inbred lines with FT being close to the nominal value of 80 days.

**Table 3:** GEBV averages of the best F_**10**_ inbred lines for the 44k rice dataset based on the index weights of 1/3, 1/3, and 1/3.

**Trait** ^1^	**Strategy** ^2^	${\bar{GEBV}}_{P}$	${\bar{GEBV}}_{F_{10}}$ ^3^
FT-Ark	GEBV-O	82.141	79.983^c^
	GD-O	84.131	79.979^c^
	GEBV-GD	84.256	79.969^c^
FT-Far	GEBV-O	72.776	74.497^d^
	GD-O	73.252	74.588^d^
	GEBV-GD	73.474	74.718^d^
FT-Abe	GEBV-O	85.435	81.797^b^
	GD-O	92.561	81.928^b^
	GEBV-GD	91.931	82.569^a^

1 *FT-Ark, flowering time in Arkansas; FT-Far, flowering time in Faridpur; FT-Abe, flowering time in Aberdeen.*

2 *GEBV-O, Subset of the 10 accessions with the highest selection index values; GD-O, Subset of the 10 accessions with the maximal D-scores chosen from the candidate set S_c_; GEBV-GD, Subset of the top two accessions with the highest selection index values, and another eight accessions chosen from the reminder of S_c_.*

3 *Different lowercase letters indicate significant differences between the strategies for a given trait (p < 0.01; LSD test)*.

The genetic gains for these nominal-the-best traits calculated using Eq. (10) are displayed in Table 4. Evidently, FT-Far undergoes a relatively smaller genetic gain among the three target traits, and the GD-O and GEBV-GD strategies lead to greater genetic gain than does GEBV-O.

**Table 4:** GEBV average for parental lines, GEBV average for F_**10**_ inbred lines, and genetic gain for the 44k rice dataset based on the index weights of 1/3, 1/3, and 1/3.

**Trait** ^1^	**Strategy** ^2^	**Mean of** $\left|{\bar{GEBV}}_{P}-\delta \right|$	**Mean of**^**3**^ $|{\bar{GEBV}}_{F_{10}}-\delta |\;$	**Genetic gain**^3^ **(nominal)**
FT-Ark	GEBV-O	2.224	0.044^a^	−2.180^d^
	GD-O	4.137	0.085^a^	−4.053^c^
	GEBV-GD	4.256	0.073^a^	−4.184^c^
FT-Far	GEBV-O	7.224	5.503^d^	−1.721^de^
	GD-O	6.748	5.412^d^	−1.336^e^
	GEBV-GD	6.526	5.282^d^	−1.244^e^
FT-Abe	GEBV-O	5.435	1.797^b^	−3.638^c^
	GD-O	12.561	1.956^c^	−10.606^a^
	GEBV-GD	11.931	2.569^c^	−9.361^b^

1 *FT-Ark, flowering time in Arkansas; FT-Far, flowering time in Faridpur; FT-Abe, flowering time in Aberdeen.*

2 *GEBV-O, Subset of the 10 accessions with the highest selection index values; GD-O, Subset of the 10 accessions with the maximal D-scores chosen from the candidate set S_c_; GEBV-GD, Subset of the top two accessions with the highest selection index values, and another eight accessions chosen from the reminder of S_c_.*

3 *Different lowercase letters indicate significant differences between the strategies for a given trait (p < 0.01; LSD test)*.

## Discussion

As suggested in Figure 1 for the tropical rice dataset, it is reasonable to infer that the secondary traits of PH and FT did not improve with the primary trait of YLD when using the single-trait selection strategies for YLD; i.e., those with the index weights of 1, 0, 0. Conversely, employing multi-trait selection strategies that used index weights of 0.6, 0.2, and 0.2 resulted in some improvement for both PH and FT. However, these results do not generally imply that grain yield will be improved more by not selecting for other traits simultaneously. There were several studies revealing that grain yield can be improved by multi-trait instead of single-trait selection. For example, selecting for grain yield and nutation content in cereal crops simultaneously (Jia and Jannink, 2012; Schulthess et al., 2016), and selecting for grain yield and yield-related traits such as harvest index, spike fertility, and thousand grain weight in wheat simultaneously (Guo et al., 2020).

**Figure 3:**
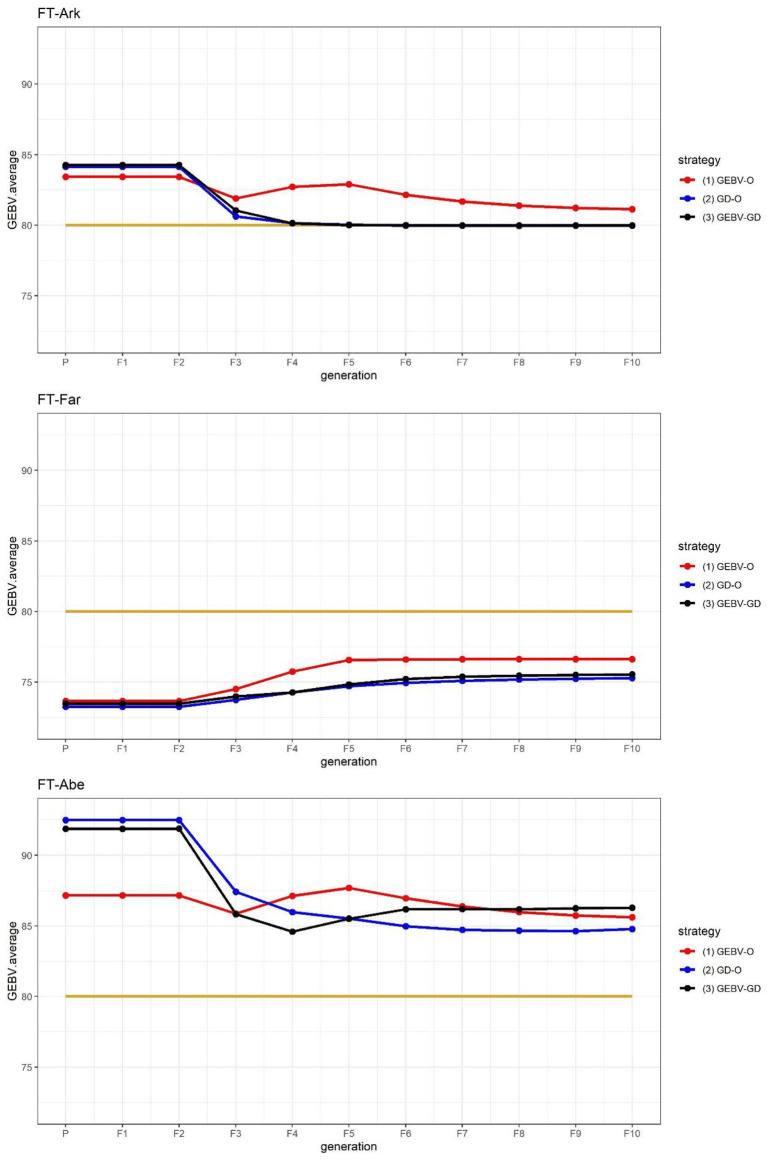
GEBV averages of the best individuals at each generation for the 44k rice dataset based on the index weights of 0.3, 0.4, and 0.3. GEBV-O, Subset of the 10 accessions with the highest selection index values; GD-O, Subset of the 10 accessions with the maximal *D*-scores chosen from the candidate set *S*_*c*_; GEBV-GD, Subset of the top two accessions with the highest selection index values, and another eight accessions chosen from the reminder of *S*_*c*_. FT-Ark, flowering time in Arkansas; FT-Far, flowering time in Faridpur; FT-Abe, flowering time in Aberdeen.

The FT at three different locations in the 44k rice dataset was used to demonstrate that the proposed approach can be applied to select parents for producing inbred lines with high adaptability to different environments. As suggested in Figure 2, the performance of FT-Far may be further improved by increasing its index weight. Hence, we modified the index weights to 0.3, 0.4, and 0.3, and re-ran the procedure; these results are displayed in Figure 3. Evidently, the curve for FT-Far got a little closer to the target value of 80 days compared with Figure 2; however, the other two traits were also affected, incurring some diminished improvement, particularly for FT-Abe. There is no golden standard for assigning the index weights to the traits because those traits can change from time to time or vary from one location to another in breeding programs (Ceron-Rojas and Crossa, 2021). Fortunately, the user can fine-tune the index weights and re-run the procedure easily using our R package, until the simulated progeny populations satisfy the desired breeding goals.

Incorporating the multi-trait GBLUP model and the selection index into the framework for single-trait selection presented in our previous article (Chung and Liao, 2020), we extended this multi-trait selection approach. Our proposed multi-trait approach was also able to conduct single-trait selection by assigning the index weight as 1 for the trait of interest, and 0 for the remaining traits. This multi-trait model-based approach is advantageous over those selected for an independent trait because it takes into account the information among the correlated traits. Moreover, our proposed approach has merit over the approach promoted by Schulthess et al. (2016), in which the selection index was treated as a new trait using a single-trait model for selection. That is, our proposed approach enabled us to assess the performance of each target trait in the progeny populations.

When conducting a breeding program to improve several quantitative traits at once, selection using a selection index is long known to be more efficient than that relying on independent culling levels or tandem selection (Hazel and Lush, 1942). Recently, Ceron-Rojas and Crossa (2021) provided a review on the statistical theory of linear selection index from phenotypic to genomic selection, in which a linear selection index was defined as a linear combination of unobservable individual traits' breeding values, weighted by the trait economic values. The proposed selection index in this study basically meets the requirements of the definition. Overall, this proposed selection index is arguably a straightforward and easy way to evaluate the composite performance of individuals. The GD quantified by the D-score is kind of different from the genetic diversity quantified by the genetic variances of traits. The former used in our study was calculated from genotypic data of individuals alone, but the latter used in the genomic usefulness function (Lehermeier et al., 2017; Yao et al., 2018) was estimated from both phenotypic and genotyped data. This means that the GD measures the genomic information for a set of individuals and is independent of the traits under investigation. Anderson et al. (1998) found that the introduction of the dominance variance has only a small positive effect on the selection response. As discussed in Ceron-Rojas and Crossa (2021), the multi-trait GBLUP model used in our study assuming that only additive effects are transmitted from generation to generation seems acceptable.

We generated the R package IPLGP to facilitate the wider application of the proposed approach. A user can install the package from the R official repository CRAN or GitHub. IPLGP provides the required R functions to replicate the results of this study. Note that a user needs to provide the linkage distances between SNP markers when running the procedure for her/his dataset. A training population consisting of both phenotypic and genotypic data is needed to build the required multi-trait GBLUP model. If historical phenotypic data are not available, a pilot experiment is recommended to phenotype a set of individuals, which can be determined using an optimization algorithm (Ou and Liao, 2019).

We addressed the crucial issue of how adequately incorporating genomic diversity into conventional truncation selection could improve the likelihood of identifying superior parental lines for multiple traits in plant breeding efforts. More importantly, we have shown that combining GP with simulated progeny populations could help breeders to discover superior parental lines before conducting field experiments. However, the phenotypic value of a trait is affected by the genotype (G), environment (E), and their G × E interaction. In reality, the local environment can significantly influence the performance of progeny populations during the growth period of each generation until they reach the F_10_ generation. As such, parental lines selected from our simulation study may not necessarily perform as expected. Therefore, conducting more field experiments with various plant species to validate our study's key findings would be worthwhile.

## Data availability statement

The original contributions presented in the study are included in the article/supplementary material, further inquiries can be directed to the corresponding author.

## Author contributions

P-YC: data curation, investigation, software, and validation. C-TL: conceptualization, project administration, supervision, writing the original draft, and review and editing. All authors contributed to the article and approved the submitted version.

## Funding

This research was supported by the Ministry of Science and Technology, Taiwan (grant number MOST 110-2118-M-002-002-MY2).

## Conflict of interest

The authors declare that the research was conducted in the absence of any commercial or financial relationships that could be construed as a potential conflict of interest.

## Publisher's note

All claims expressed in this article are solely those of the authors and do not necessarily represent those of their affiliated organizations, or those of the publisher, the editors and the reviewers. Any product that may be evaluated in this article, or claim that may be made by its manufacturer, is not guaranteed or endorsed by the publisher.
